# The development and application of audit criteria for assessing knowledge exchange plans in health research grant applications

**DOI:** 10.1186/s13012-014-0093-0

**Published:** 2014-07-14

**Authors:** Alexa I Ruppertsberg, Vicky Ward, Alicia Ridout, Robbie Foy

**Affiliations:** 1 Health Services Innovation Hub, Faculty of Medicine and Health, University of Leeds, Leeds, UK; 2 Leeds Institute of Health Sciences, University of Leeds, Leeds, UK

**Keywords:** Knowledge exchange, Impact plans, Research proposals

## Abstract

**Background:**

Research funders expect evidence of end user engagement and impact plans in research proposals. Drawing upon existing frameworks, we developed audit criteria to help researchers and their institutions assess the knowledge exchange plans of health research proposals.

**Findings:**

Criteria clustered around five themes: problem definition; involvement of research users; public and patient engagement; dissemination and implementation; and planning, management and evaluation of knowledge exchange. We applied these to a sample of grant applications from one research institution in the United Kingdom to demonstrate feasibility.

**Conclusion:**

Our criteria may be useful as a tool for researcher self-assessment and for research institutions to assess the quality of knowledge exchange plans and identify areas for systematic improvement.

## Background

The recognised gap between healthcare research and practice has led to research funders, amongst other initiatives, introducing explicit expectations that applications detail the expected impact of research and demonstrate how it will be achieved [[1]–[3]]. Similarly, the UK’s Research Excellence Framework now includes a retrospective evaluation of the economic, societal and cultural ‘impact’ of University-based research as well as its scientific quality [[4]]. There are a number of resources to help researchers think about how best to increase the impact of their research and write knowledge exchange plans [[5]–[8]]. Noting that Tetroe *et al.* identified up to 29 terms used for ‘knowledge transfer’ [[1]], we use the term ‘knowledge exchange’ to describe the multidirectional, dynamic and iterative nature of translating research-based knowledge into policy and practice [[9]]. Knowledge exchange encompasses a number of activities, such as dissemination (*i.e.*, sharing of research findings), collaboration and consultancy, which ideally result in a range of impacts.

Resources are available to help reviewers assess knowledge exchange plans [[2]]. Despite the presence of such guidance and funders’ insistence that research proposals include explicit knowledge exchange and impact plans, a general standard for assessing those plans is not readily available. Funders provide guidelines to reviewers, which vary significantly across funders and funding programmes. Some of these guidelines are freely available whilst others are only made available to reviewers directly when asked to review a proposal. It can therefore be difficult for researchers to know whether reviewers are likely to judge their knowledge exchange plans as suitable. Furthermore, it is rare for researchers to receive feedback on this aspect of their proposal. This is unsurprising since these plans currently form a relatively limited part of the assessment process. Indeed, if researchers have followed recent advice to embed knowledge exchange principles and mechanisms throughout the entire lifecycle of a research project [[2],[5]], it may be relatively difficult for reviewers to directly comment on this aspect of a proposal. This situation offers little scope for researchers to learn about how knowledge exchange can be better incorporated into the research process. There is a risk that researchers will come to see knowledge exchange and impact plans as a ‘tick-box’ exercise rather than considering how these could genuinely improve the entire research project.

Having been involved in advising a number of colleagues in our own institution on how to enhance knowledge exchange plans, we began to consider how to change researcher behaviour. We therefore took an approach based upon the principles of audit and feedback [[10]]. We developed criteria for assessing knowledge exchange plans within research proposals that could be applied to a sample of grant proposals and fed back to researchers. We demonstrate the feasibility of criteria development and assessment before discussing their potential to help researchers to improve their knowledge exchange plans.

## Findings

### Criteria development

We aimed to develop assessment criteria that drew upon existing conceptual frameworks, were underpinned by a sound rationale, and could potentially be measured from a review of written grant proposals. We extracted candidate knowledge exchange principles and recommendations from a review and synthesis of knowledge exchange frameworks, supplemented by existing guidance issued by UK research councils [[6]-[8],[11]]. Following iterative development, including feedback from academic colleagues, we established a set of 19 criteria for assessing knowledge exchange plans grouped under five thematic headings (Table 1). The five themes cover: problem definition; involvement of research users; public and patient engagement; dissemination and implementation; and planning, management and evaluation of knowledge exchange.

**Table 1:** Criteria for the assessment of knowledge exchange plans and illustrative text from proposals

**Themes and criteria**	**Illustrative anonymised text from grant proposals**
*Problem definition*	
There is a statement about the problem addressed by this proposal and its significance to the health service or health.	Around 150,000 people each year attend hospitals in England due to self-harm, many of them more than once. Over 5,000 people die by suicide each year in the UK, a quarter of them having attended the general hospital in the previous year because of non-fatal self-harm, so self-harm is the major identifiable risk factor for suicide. People receive a variable standard of care at hospital: many are not assessed for their psychological needs, and little psychological therapy is offered. Despite its frequent occurrence, we have no clear research evidence about how to reduce repetition of self-harm. People who have self-harmed show less active ways of solving problems, and brief problem-solving therapies are considered the most promising psychological treatments.
There is a statement about how the problem has been identified.	Routinely collected data show a sustained but poorly understood increase in primary care prescribing of opioids. We conducted an online survey of pharmacy advisers and found that 88% were concerned about current patterns of opioid prescribing for chronic, non-cancer pain whilst 95% considered opioids were inappropriately prescribed.
The proposal identifies specific users of the research.	The main beneficiaries will be all those interested in reducing smoking initiation in adolescents. This includes teachers and those interested in promoting health in adolescent samples. Others will include academic and other beneficiaries such as social scientists and health researchers interested in understanding behaviour change in adolescent samples.
There is a clear description of user benefit(s).	Outcomes from this research will be instrumental in determining the implementation of laparoscopic rectal cancer surgery throughout the UK National Health Service (NHS) and the rest of the world. It will provide information to clinicians in terms of which patients might benefit from a laparoscopic approach, to healthcare providers in determining cost-effectiveness, and to patients to inform personal choice. It will inform government agencies responsible for issuing guidance and strategy and provide the evidence for future clinical initiatives.
*Involvement of research users*	
There is evidence that users have been actively engaged in the development of this proposal.	Following our synthesis of current literature, we consulted with our local network of family doctors. Of the potential options to improve the safety of prescribing practice, they expressed a preference for computerised prompts but stipulated that the alert messages should only be triggered in specific circumstances.
There is a plan as to how users will be involved in the conduct or management of the research.	We will work with and seek feedback from a stakeholder panel following each step of intervention development. The panel will comprise health service managers and clinicians and advise upon the feasibility, acceptability and sustainability of candidate intervention components within available resources and systems.
There is a plan as to how users will be involved in the dissemination and implementation of research findings.	Our implementation field manual will be developed through structured exchanges with relevant groups and advisors, including the National Institute for Health and Care Excellence (NICE), members of clinical commissioning groups and clinical governance leads. The manual will be developed iteratively from the start of the research programme. We will share our plans and emerging findings and seek feedback to refine our interpretation and methods.
*Patient and public engagement*	
There is evidence of patient or public engagement in the development of this proposal.	The project was discussed with consumer colleagues during the development phase. The concept of the research was supported. We recognise that the proposed study presents important ethical issues, particularly whether or not to inform participants of their test results. The clear advice from our consumer group, summarised in their report to us entitled ‘To tell or not to tell’, was the dominant view that all participants should be treated as responsible individuals and that each person should be free to decide what information they wish to receive.
There is a plan as to how patients or the public will be involved in the conduct or management of the research.	Service users will play a vital role in helping guide this project to a successful conclusion. X (co-applicant) has been involved in discussions throughout the development of this proposal and will continue to play an active role in the project, as a full member of the project and steering committees. In addition, we intend to recruit a small, service user advisory group to provide active input at specific points throughout the project. Y will facilitate the group and provide mentorship and support to its members. Members will be asked to provide input in relation to recruitment and retention, study materials (participant information sheet, consent form), design of the topic guide for the qualitative study, and provide feedback on the analysis of the qualitative data.
There is a plan as to how patients or the public will be involved in the dissemination and implementation of research findings.	Our Older People’s Forum is well established and has been provided with training and support as required through their development. Quarterly progress reports to the Forum will be a catalyst for discussion of concerns/queries or comments about the research and to inform dissemination strategies.
*Dissemination and implementation*	
The proposal describes the rationale(s) behind the selection of dissemination and implementation strategies.	We recognize the barriers faced when trying to embed new service developments into routine care. We will therefore develop a model service specification that also sets out expected outcomes and delineates the necessary resources and training required to bring about a change in service delivery.
The proposal describes how the dissemination and implementation strategies build upon existing resources and ways of working in a sustainable manner.	If proven cost-effective, our computerized prompts can be readily incorporated within or adapted to existing clinical information systems.
There is a statement about how the research process and findings will be used.	The study outputs will: inform the development of guidelines by identifying unmet needs in chronic, non-cancer pain and potential opportunities during patient trajectories to intervene (*e.g.*, medication reviews); target prescribing advisors, who will therefore be able to provide better-informed education and support to practices in challenging or changing opioid prescribing; and inform commissioning of pain services, especially if our findings suggest greater scope for specialist services in developing shared care plans for patients that emphasise active clinical review and non-pharmacological interventions.
Networks for sharing findings with users are clearly identified.	Theoretical and practical outcomes of the study will be presented to patients, carers, and members of Parkinson’s UK at a workshop and open day. Public awareness of this work will be raised at events such as Café Scientifique and Brain Awareness Week and through the University’s press office.
There is a plan for ensuring that any disseminated materials and products are tailored to the needs of users.	The Cancer Patients and Public in Research group has agreed to help structure content and dissemination strategy of the research results. They will provide input in the lay summary to ensure that the results will be presented in the appropriate way to patients and the lay public; they will provide advice on additional ‘non-academic’ dissemination of the project results (be that newsletters in patient cancer journals or local or national cancer network websites).
*Planning, management and evaluation of knowledge exchange*	
The timing and order of knowledge exchange activities is stated.	We will have an open day near the end of the project for the Parkinson’s Disease Society members to see our findings. We have regularly organised such events for our older adult participants (with over 100 attendees). We will also hold a workshop during the final year with 10 Society members to obtain feedback on the proposed methods to discuss whether they would be beneficial in their everyday lives. Finally, we will send two newsletters a year to our participants to inform about progress. We already send newsletters to our older adult participants, and they find this a rewarding part of the process.
The resources required for knowledge exchange (including people, services and consumables) have been identified (whether or not the proposal seeks funding for them).	We are seeking funding to cover the costs of our dissemination activities, including for the design and printing of a briefing for service commissioners and the development of an interactive website for patients and clinicians. We also seek funding to pay for the costs of an open-access journal publication.
Applicants’ previous experience of undertaking knowledge exchange activities is described.	Our academic unit has a strong track record of delivering continuing professional development courses for clinicians. We will add a new module, informed by this programme of research, to our existing suite of courses, which we anticipate running twice a year.
The proposal states ways in which the uptake of research findings can be monitored.	The patient outcomes measured as part of this trial have been designed to be derived from existing routinely recorded clinical data. Therefore, they can be applied to monitoring the implementation of the trial treatment in routine practice following trial completion.

### Application of the assessment criteria

We applied the criteria in an audit of applied health research proposals submitted from our own institution. We designed each criterion so that it could be rated as ‘met’ (scoring ‘1’) or ‘not met’ (scoring ‘0’) from reviewing grant proposals. We also anticipated that judgements upon whether or not each criterion was met would depend upon an assessment of the entire proposal as opposed to only ‘dissemination plans’ or equivalent. We took this approach because we expected evidence of knowledge exchange to be embedded throughout proposals (*e.g.*, ‘problem definition’ in introductory sections). Three project team members (AIR, AR and RF) piloted the criteria by independently assessing three proposals, comparing assessments, and then clarifying criteria where necessary.

We screened the titles of grant proposals recorded by the Faculty of Medicine and Health, University of Leeds, which were submitted between May 2011 and May 2012. We selected 102 with a likely focus on applied health research. We included pending, successful and unsuccessful proposals because we sought a representative range of applications. We subsequently identified 25 full proposals led by academics in our institution. The majority of these were submitted to various National Institute of Health Research (NIHR) programmes (20), three to UK research councils, and two to other funders. We obtained permission from all lead applicants to review their grant applications in full. One project team member (AR) then applied the criteria to each application.

**Figure 1:**
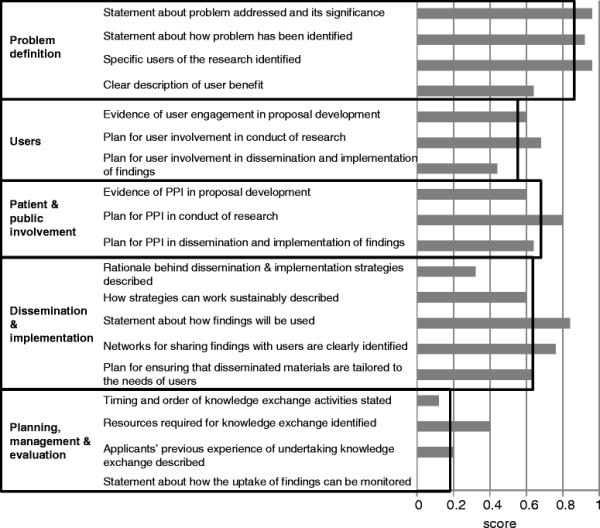
Mean criterion scores (grey bars) and mean themes scores (black outline bars) across the 25 proposals in the audit.

We calculated mean scores for each criterion and also for each theme across the 25 proposals (Figure 1). Proposals scored highest in problem definition (0.87, out of a maximal 1), followed by public and patient involvement (0.68), dissemination and implementation (0.63), and involvement of users (0.57), and lowest in planning, management and evaluation of knowledge exchange activities (0.18).

Amongst individual criteria, the three most frequently met were: ‘problem addressed by this proposal and its significance to the health service or health is stated’ (24 of 25 proposals); ‘specific users of research are identified’ (24 proposals); and ‘statement about how the problem has been identified’ (23 proposals). The three least frequently met criteria were: ‘ways in which the uptake of research findings can be monitored’ (no proposals); ‘timing and order of knowledge exchange activities is stated’ (3 proposals); and ‘applicants’ previous experience of undertaking knowledge exchange activities is described’ (5 proposals). A mean of 11.2 criteria out of a maximal 19 were met across the proposals (range 5.8 to 16.3). Table 1 also illustrates part-anonymised text from proposals that would allow a criterion to be judged as met (with the addition of a fictional example for one criterion met by no applications).

## Conclusions

It is feasible to develop and apply audit criteria for assessing knowledge exchange plans within research proposals. We suggest they can be used by individual researchers and teams for self-assessment, or by grant-seeking institutions to identify common strengths and weaknesses and hence guide staff development. Our modest analysis of one institution suggests some key challenges that others are likely to face, especially around identifying resources and methods to monitor the longer term impact of research.

Developing meaningful and feasible criteria posed three main challenges. First, we aimed to develop criteria that would offer researchers enough detail to guide improvement of knowledge exchange plans whilst avoiding over-specification. We found it helpful to organise the emerging criteria into five themes that both followed the flow of a proposal and strongly related to the knowledge exchange process [[9],[11]]. The themes helped to contextualise the criteria and safeguarded against missing aspects of the knowledge exchange process. Second, there is a risk that rather than encouraging a longitudinal view of the knowledge exchange process, our audit criteria may promote a tokenistic ‘box-ticking’ approach by applicants [[2],[5]], especially if their institutions use measurement as a feature of performance management [[12]]. Any audit instrument is prone to the same misuse and degrees of self-deception. Furthermore, developing and stating a plan for knowledge exchange is more likely in principle to result in action than not making a plan [[13]]. Third, we were aware of the need to capture knowledge exchange plans aimed at a range of different research ‘users’. The Canadian Institutes of Health Research (CIHR), for instance, explains that ‘A knowledge user can be, but is not limited to, a practitioner, a policy maker, an educator, a decision maker, a health care administrator, a community leader or an individual in a health charity, patient group, private sector organization or media outlet’ [[14]]. The UK NIHR states that ‘the term user refers to patients, their carers and family members, as well as to members of the public and representatives from patient and charitable organisations’ [[15]]. We therefore distinguished between immediate users of research findings (*e.g.*, clinicians, commissioners) and longer-term beneficiaries (*e.g.*, patients).

Applying the tool to research proposals also posed a number of challenges. First, funders have adopted different concepts of knowledge exchange and impact, and use different terminology [[1]]. We suggest that our criteria are sufficiently generic to be transferable beyond the funding applications we assessed from one UK institution. Second, proposal forms differ substantially across different funders and programmes, making it necessary for assessors to read entire proposals to capture the full extent of knowledge exchange plans. Third, some criteria within the planning, management and evaluation of knowledge exchange theme scored poorly; *e.g.*, none of the 25 proposals included a statement about the monitoring of the uptake of research findings. This may reflect both the absence of explicit guidance by funders and limited experience and skills amongst researchers. Fourth, researchers and institutions will inevitably raise the question of whether stronger knowledge exchange plans actually enhance the chances of grant success. We did not examine associations with success, partly because of the small number of applications reviewed but mainly because this was not the key aim. Whilst demonstrating stronger knowledge exchange may have variable impacts upon the likelihood of success, we suggest that the fundamental issue concerns how to maximise the chances of relevant impact during and following research projects.

In summary, research funders and institutions are increasingly interested in demonstrating impact. Researchers are therefore expected to present clear knowledge exchange plans, ideally embedded throughout the whole research cycle. We suggest that our criteria are useful for researcher self-assessment of individual applications and as an audit tool for research institutions to identify areas for improvement. Given the limited, exploratory nature of this work, we welcome further suggestions and debate around how to enhance the validity and relevance of such audit criteria.

## Abbreviations

NIHR: National Institute of Health Research

NICE: National Institute for Health and Care Excellence

NHS: National Health Service

UK: United Kingdom

## Competing interests

The authors declare that they have no competing interests.

## Authors’ contributions

All authors contributed to the criteria development. AIR, AR and RF piloted the criteria, and AIR coordinated author permissions and retrieved and assessed all applications using the criteria. AIR, VW and RF drafted the manuscript and table, and AIR produced the figure. RF conceived of the study. All authors read and approved the final manuscript.
